# Koilocytosis in LSIL Cytology Has Limited Predictive Value for CIN2+ in HPV-Positive Women: Implications for Risk-Based Cytology Triage

**DOI:** 10.3390/pathogens15050537

**Published:** 2026-05-15

**Authors:** Yukimi Misawa, Shuichi Mizuno, Saeka Honda, Ruku Shinohara, Koki Kikuchi, Rei Settsu, Kaori Okayama, Masahiko Fujii, Mizue Oda, Mitsuaki Okodo

**Affiliations:** 1 Department of Medical Technology, Faculty of Health Sciences, Kyorin University, 5-4-1 Shimorenjaku, Mitaka-shi 181-8621, Japan; yukimi.misawa@yamatokai.or.jp (Y.M.); mizuno1911h@std.kyorin-u.ac.jp (S.M.); honda2421n@std.kyorin-u.ac.jp (S.H.); shinohara2111h@std.kyorin-u.ac.jp (R.S.); kikuchi2211h@std.kyorin-u.ac.jp (K.K.); rei-settsu@ks.kyorin-u.ac.jp (R.S.); 2 School of Health Sciences, Department of Medical Technology and Sciences, International University of Health and Welfare, 2600-1 Kitakanemaru, Otawara-shi 324-8501, Japan; okayaman0811@std.kyorin-u.ac.jp; 3 Department of Clinical Laboratory, Genki Plaza Medical Center for Health Care, 1-7-1 Jinbocho, Chiyoda-ku, Tokyo 101-0051, Japan; fujiim1951-1011@tbz.t-com.ne.jp; 4 Department of Gynecology, Genki Plaza Medical Center for Health Care, 1-105 Jinbocho, Chiyoda-ku, Tokyo 101-0051, Japan; m-oda@genkiplaza.or.jp

**Keywords:** cytology, human papillomavirus, koilocytosis, LSIL, CIN2+, cervical cancer screening, HPV genotyping, cytology triage, risk stratification, productive HPV infection

## Abstract

Cervical cancer screening with high-risk human papillomavirus (HR-HPV) testing requires effective triage of HPV-positive women. Koilocytosis is a classic cytopathic effect of HPV infection, but its clinical significance in low-grade squamous intraepithelial lesions (LSILs) remains unclear. We retrospectively evaluated 157 HPV-positive women with LSIL cytology and follow-up data, including 140 women with concurrent biopsy results. Koilocytes were identified in 93/157 cases (59.2%) and were less frequent in HPV16/18-positive cases. Cervical intraepithelial neoplasia ≥ grade 2 (CIN2+) was detected in 9/84 koilocyte-positive cases (10.7%) and 16/56 koilocyte-negative cases (28.6%), whereas non-CIN findings were more common in koilocyte-positive cases. Koilocyte-positive cases also showed a longer time to regression from LSIL to negative for intraepithelial lesions or malignancy. These findings suggest that koilocytosis mainly reflects productive HPV infection and has limited utility for predicting CIN2+ in HPV-based screening triage. Excluding koilocytosis-driven low-grade cytological changes from triage positivity criteria may improve specificity and positive predictive value, supporting higher triage thresholds.

## 1. Introduction

High-risk human papillomavirus (HR-HPV) is a well-established cause of cervical cancer [1,2]. In recent years, many countries have adopted primary screening strategies based on HPV testing alone [3,4]. Although HPV testing is more sensitive to cervical intraepithelial neoplasia grade 2 or more (CIN2+), its specificity is limited [3,5]. Therefore, current screening guidelines recommend cytology-based triage to guide further management of HPV-positive individuals [4,6]. A threshold of atypical squamous cells of undetermined significance (ASC-US) or worse is commonly used to select patients for further diagnostic evaluation. This aims to ensure the detection of clinically significant lesions while minimizing unnecessary colposcopies [7]. However, ASC-US and low-grade squamous intraepithelial lesion (LSIL) are frequently associated with inflammation, reparative changes, and transient HPV infections, and the prevalence of CIN3+ in these patient populations is low [4,8,9]. Previous studies have shown that among HPV-positive women, ASC-US and LSIL confer relatively similar CIN2+/CIN3+ risks, supporting similar clinical management strategies [10,11]. Consequently, HPV-based primary screening increases the proportion of false-positive cytology results, reducing the specificity of cytology as a triage tool [3,4,6,12,13].

To address this issue, risk stratification based on HPV genotyping has gained increasing attention. As individuals positive for HPV 16/18 are known to have a high risk of CIN3+, some countries have adopted a policy of direct referral for colposcopy without cytology triage with these HPV types, including the United States and Australia [6,8,9,14]. However, infection with other high-risk HPV types is often transient, and their clinical significance is more heterogeneous. This poses challenges for appropriate risk stratification and the avoidance of unnecessary referrals [4,8,9,15].

To maintain a high positive predictive value (PPV), Australia, where HPV vaccination coverage is high, has raised the cytology triage threshold to atypical squamous cells, which cannot exclude high-grade squamous intraepithelial lesions (HSILs) (ASC-H) or worse [16]. This strategy includes monitoring of ASC-US and LSIL cases. If HPV-negativity is confirmed at 12 months, there is no clinical intervention [16]. This places greater emphasis on the persistence of HPV infection than on morphological abnormalities. In contrast, many countries, including Japan, suffer from limitations such as low vaccination coverage, long screening intervals, insufficient follow-up rates, and no nationwide tracking system. This has led to the continued use of a fail-safe approach that prioritizes sensitivity, with ASC-US or worse considered positive [4,12,17,18]. Raising the triage threshold to ASC-H or worse requires both robust cytological evidence of negative ASC-US and LSIL and system-level assurance of their identification.

Koilocytosis is a well-recognized cytological feature of LSIL and may occasionally contribute to ASC-US interpretations [19]. Most women with definite koilocytic changes in cervical cytology are expected to have a positive HPV test, consistent with the interpretation of koilocytosis as a cytopathic effect of productive HPV infection. The Bethesda system defines koilocytes as squamous epithelial cells with well-defined perinuclear halos (cytoplasmic vacuolization), accompanied by mild nuclear enlargement and irregularity. They are regarded as a cytopathic effect of HPV infection [19]. The perinuclear halo is thought to reflect the cytoplasmic alterations associated with viral replication [15,19]. However, recent studies suggest that koilocytosis is not uniformly associated with all HPV types, but occurs more frequently in non-16/18 high-risk HPV types and low-risk HPV types [20]. The associated changes are often transient and tend to regress spontaneously [15]. Furthermore, koilocytosis may be mimicked by inflammatory changes or glycogen-related perinuclear clearing, leading to interobserver variability in its identification [19,21]. In addition, previous studies have demonstrated considerable interobserver variability in cytology-based cervical cancer screening interpretations, particularly in low-grade cytological abnormalities [22].

Currently, cytology-based triage relies primarily on diagnostic categories such as ASC-US and LSIL. Although koilocytosis is widely recognized as a cytopathic effect of productive HPV infection and a characteristic feature of LSIL [19], its independent utility for predicting CIN2+ in HPV-positive women remains unclear. Therefore, its inclusion as a characteristic of ASC-US and LSIL may contribute to reduced specificity and unnecessary referrals.

In light of this, the re-evaluation of koilocytosis as an independent morphological feature in HPV-based primary screening may help identify cases in which ASC-US and LSIL can be safely considered negative. The interpretation of koilocytosis as an infection-related cytological change may improve risk stratification and support the adoption of higher triage thresholds, such as ASC-H.

This study aims to clarify the clinical significance of koilocytosis in cytology-based triage by analyzing its association with HPV genotype distribution, concurrent histological findings, and longitudinal clinical outcomes. In this study, we specifically focused on HPV-positive women with LSIL cytology, rather than ASC-US, to evaluate the clinical significance of koilocytosis within a morphologically defined low-grade lesion category.

## 2. Materials and Methods

### 2.1. Clinical Samples

This retrospective single-center study included cervical samples collected by a single experienced gynecologist at the Genki Plaza Medical Center between 2014 and 2025. Restricting the cohort to samples collected by a single physician minimized variability in sampling procedures and specimen quality. During the study period, 787 women underwent HPV testing, cervical cytology, and biopsy evaluation within this standardized clinical setting. Among these, 157 women who were HPV-positive, diagnosed with LSIL on Pap smear at the initial visit, and had available follow-up data were included in the study.

The mean (range) age was 37 (21–68) years. The mean (range) number of cytology examinations was 5.2 (2–18). The mean (range) follow-up duration was 18 (1–78) months. All cervical samples were collected by a single gynecologist using SurePath™ liquid-based cytology (LBC) (Becton, Dickinson & Co., Franklin Lakes, NJ, USA). Colposcopy-directed biopsies were performed immediately after LBC sample collection. The study protocol was approved by the Kyorin University Institutional Review Board (approval no. 2023-1-1), and all procedures were conducted in accordance with the 1964 Declaration of Helsinki and its later amendments.

### 2.2. Cytology and Histology

From each LBC sample, we prepared two smear slides. These were fixed in 95% ethanol and stained using the Pap method [19]. All Pap smears were independently evaluated by five cytotechnologists using the Bethesda system [16]. The biopsy specimens were fixed in neutral-buffered formalin and embedded in paraffin. Sections were stained with hematoxylin and eosin and evaluated for CIN.

### 2.3. Assessment of Koilocytes and Non-Koilocytotic Cells in Pap Smear Specimens

**Figure 1:**
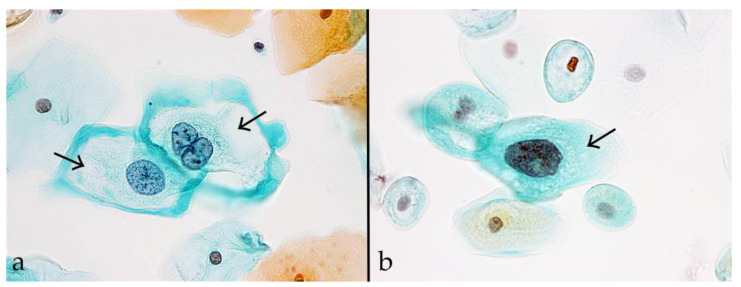
Representative images of koilocytes and non-koilocytotic cells in Pap smears. (**a**) Koilocytes are large squamous epithelial cells corresponding to low-grade squamous intraepithelial lesions (LSILs). They are characterized by distinct perinuclear halos (cytoplasmic vacuolization) and nuclear atypia. The arrows indicate the perinuclear halos. (**b**) Non-koilocytotic cells are LSIL-type cells that lack cytoplasmic vacuolization. The arrows indicate the cells without perinuclear halos. Magnification ×40.

Koilocytes were defined as large squamous epithelial cells corresponding to LSIL, with cell diameters ranging between approximately 35–50 µm. The Bethesda system indicates that these cells exhibit distinct perinuclear halos (cytoplasmic vacuolization) and nuclear atypia (Figure 1a) [19]. Non-koilocytotic cells were defined as LSIL-type cells lacking cytoplasmic vacuolization (Figure 1b). The samples were independently assessed by five cytotechnologists for the presence of koilocytes. Cases were classified as koilocyte-positive when at least three of the cytotechnologists identified one or more definite koilocytes. All other cases were classified as koilocyte-negative.

### 2.4. Human Papillomavirus Genotyping of Whole Liquid-Based Cytology Samples, Micro-Dissected Cells, and Biopsy Specimens

For HPV genotyping of whole LBC samples, we obtained approximately one-tenth of the cell pellet obtained from each residual LBC sample. This was lysed in 100 μL of alkaline lysis buffer (25 mM sodium hydroxide [NaOH] and 0.2 mM ethylenediaminetetraacetic acid [EDTA], pH 12.0) [23]. The target cells were isolated from Pap smears by microdissection [24] and lysed in 20 μL of the same buffer. The two 5 µm thick paraffin sections previously fixed from the biopsy samples of each patient were deparaffinized and lysed in 50 µL of alkaline solution. All lysates were neutralized with an equal volume of 0.04 M Tris-HCl (pH 5.0) and centrifuged at 13,200 revolutions per minute for 1 min. They were then used directly as deoxyribonucleic acid (DNA) templates for polymerase chain reactions (PCRs) without quantification.

HPV genotyping was performed using a highly sensitive type of PCR known as uniplex E6/E7 PCR [25]. This assay detects 14 high-risk HPV types (HPV 16, 18, 31, 33, 35, 39, 45, 51, 52, 56, 58, 59, 66, and 68), as well as multiple other HPV types, including HPV 6, 11, 26, 30, 34, 40, 42, 44, 53, 54, 55, 61, 62, 67, 69, 70, 71, 73, 74, 81, 82, 84, 85, and 90. The latter includes low-risk varieties as well as types of uncertain or potential oncogenic risk. The detection limit is approximately 100 copies, and no cross-reactivity between genotypes has been observed. Using PCR amplification of a 110 bp fragment of the β-globin gene with PC03/PC04 primers (Takara Bio, Shiga, Japan), we began by confirming DNA quality. Subsequently, we performed individual PCR amplification of each HPV genotype under standardized conditions. The PCRs were performed in a total volume of 10 μL containing 1× AmpliTaq Gold^®^ 360 buffer (Applied Biosystems, Foster City, CA, USA), 2 mM MgCl_2_, 0.025 U/μL AmpliTaq Gold 360 DNA polymerase, 2 μL of DNA template, and 0.5 pmol primers. Amplification was performed using a T100™ thermal cycler (Bio-Rad Laboratories, Tokyo, Japan), with 40 cycles of denaturation at 95 °C for 30 s, annealing at 60 °C for 30 s, and extension at 72 °C for 30 s, including an initial 10 min denaturation step and a 5 min final extension step. The negative controls included an HPV-negative HL60 cell lysate for HPV assays and DNA-free water for β-globin. The positive controls included plasmid DNA containing full-length HPV genomes and HL60 cell lysate for β-globin amplification. β-globin amplification was not performed on DNA extracted from micro-dissected single cells.

### 2.5. Statistical Analysis

All statistical tests were performed using SPSS for Windows, v.25.0 (IBM Corp., Armonk, NY, USA). We evaluated the relationships between koilocyte status (presence vs. absence), HPV infection status (single vs. multiple infection), and HPV genotype group (HPV 16/18, other high-risk types, and non-high-risk HPV types [including low-risk types and types with uncertain oncogenic potential], and a predefined group of 11 koilocytosis-associated HPV types [HPV 39, 56, 66, 6b, 40, 42, 53, 61, 74, 89, and 90]) [16] using chi-square or Fisher’s exact test. In cases with multiple HPV infections, each sample was assigned to a single HPV genotype group using hierarchical classification based on oncogenic risk. Priority was given to HPV 16/18, followed by other high-risk types, and then non-high-risk HPV types. *p*-values < 0.05 were considered statistically significant.

We analyzed the association between koilocyte status and histological CIN grade in the 140 cases that underwent simultaneous biopsy (the remaining 17 cases had no concurrent biopsy data). We assessed HPV genotype concordance between whole LBC samples and biopsy specimens. Concordance was defined as the presence of at least one identical HPV genotype in both samples. To evaluate the origin of atypical cells, we compared the HPV genotypes of micro-dissected koilocytotic and non-koilocytotic cells with those of the corresponding biopsy specimens. Agreement was quantified using Cohen’s kappa coefficient with 95% confidence intervals (CIs).

We used the Kaplan–Meier method to analyze the time to regression from LSIL to negative for intraepithelial lesions or malignancy (NILM) and calculated the median regression time. Differences between groups were assessed using log-rank tests, with *p*-values < 0.05 considered statistically significant.

## 3. Results

**Figure 2:**
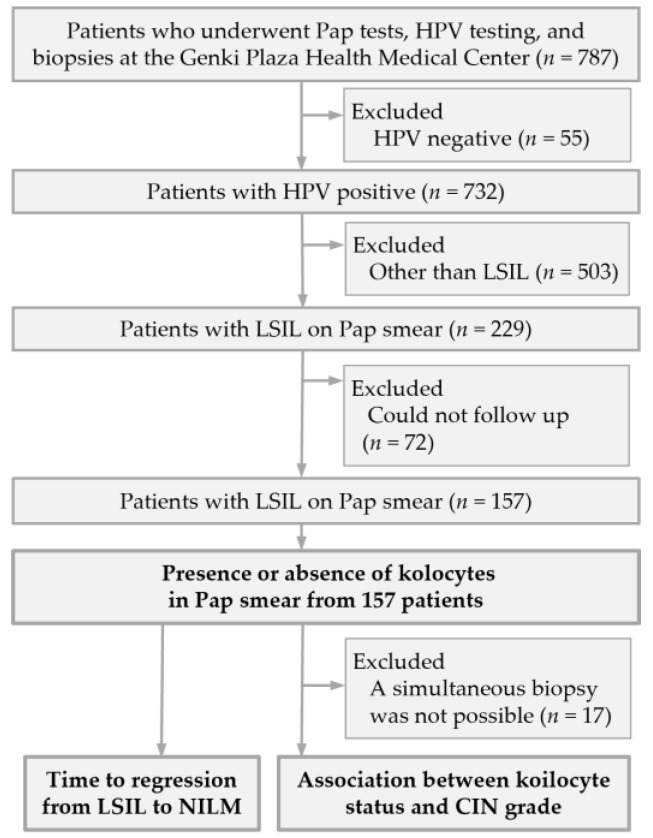
Flow diagram of patient selection for the evaluation of koilocytes in Pap smear specimens.

A total of 787 women underwent gynecological screening during the study period. Among these, 157 HPV-positive women with LSIL cytology and available follow-up data were included in the study. The remaining 630 women were excluded, including 55 HPV-negative cases, 503 cases without LSIL cytology, and 72 cases without available follow-up data (Figure 2).

The clinicopathological and HPV genotyping data of individual cases are provided in Table S1. Koilocytes were detected on the Pap smears of 93 of the 157 (59.2%) LSIL cases. Multiple HPV infections were identified in 124 of the 157 (79.0%) cases. Koilocytes were present in 70 (56.5%) and absent in 54 (43.5%) of these 124. We found no significant association between koilocytosis and HPV infection status (*p* = 0.232).

Koilocytes were present in 12 (40.0%) of the 30 patients with HPV 16/18, 67 (61.5%) of the 109 with other high-risk HPV types, and 14 (77.8%) of the 18 with non-high-risk HPV types. We found a significant association between koilocyte presence and HPV genotype group (χ^2^ (2) = 7.39, *p* = 0.025, Cramer’s V = 0.217) (Table 1). Residual analysis revealed that koilocytes were significantly less frequent in HPV 16/18-positive cases (*p* = 0.017).

Koilocytes were detected in 79 (84.9%) cases with koilocytosis-associated HPV types and in 40 (62.5%) with non-koilocytosis-associated HPV types. The odds of detecting koilocytes were significantly higher in the koilocytosis-associated HPV types group than in the non-koilocytosis-associated HPV types group (odds ratio 3.39, 95% CI: 1.58–7.25, *p* = 0.002).

The relationships between the presence of koilocytes and histological findings are shown in Table 2. After excluding the 17 cases without concurrent biopsy results, 140 cases were analyzed. We found koilocytes in 38 (73.1%) non-CIN cases, 37 (58.7%) CIN1 cases, and nine (36.0%) CIN2+ cases. We found a significant association between koilocyte presence and histological diagnosis (χ^2^ (2) = 9.75, *p* = 0.008, Cramer’s V = 0.264). Residual analysis revealed that koilocytes were significantly more frequent in non-CIN cases (*p* = 0.015) and significantly less frequent in CIN2+ cases (*p* = 0.007). Conversely, koilocyte-negative cases were less frequent in non-CIN and more frequent in CIN2+ lesions (Table 2).

Next, we evaluated HPV genotype concordance between whole LBC samples and the corresponding biopsy specimens. The concordance rates were 75.0% (63/84) in koilocyte-positive cases and 80.4% (45/56) in koilocyte-negative cases, with no significant difference between the two groups. Among the discordant cases, the proportion of HPV-negative biopsy specimens was 71.4% (15/21) in koilocyte-positive cases and 63.6% (7/11) in koilocyte-negative cases. The remaining cases showed discordance between the HPV genotypes identified by cytology and histology. To further investigate the origin of atypical cells, we isolated koilocytotic and non-koilocytotic cells from Pap smears by microdissection. We then compared their HPV genotypes with those of the corresponding biopsy specimens. After excluding 84 cases in which no HPV was detected at the single-cell level (classified as not evaluable due to lack of β-globin amplification), 56 cases remained. The HPV genotypes detected in single cells were consistent with those in the corresponding LBC samples. However, we found concordance between single cells and biopsy specimens in only 33 cases (58.9%), indicating moderate agreement (Cohen’s κ = 0.471).

**Figure 3:**
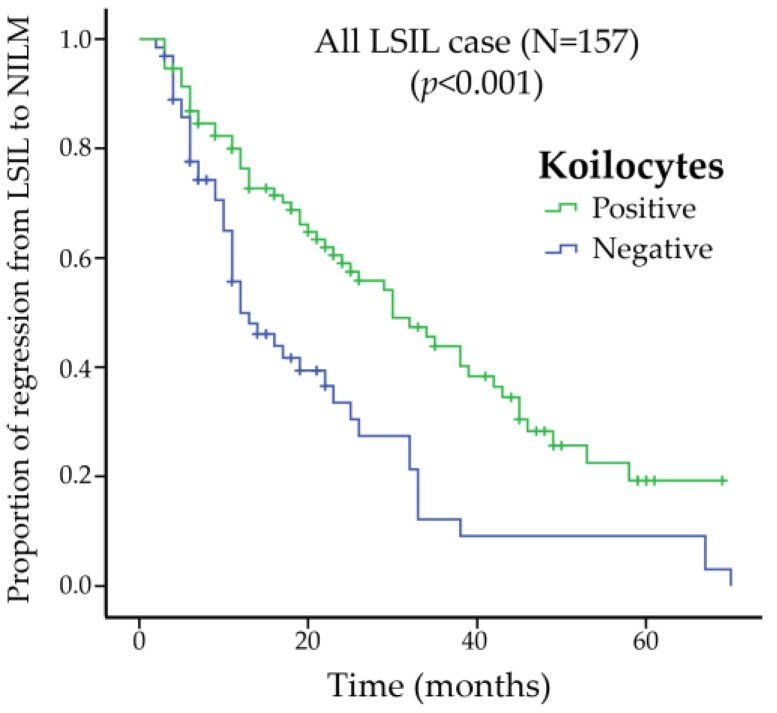
Kaplan–Meier analysis of time to regression from low-grade squamous intraepithelial lesion (LSIL) to negative for intraepithelial lesions or malignancy (NILM) according to koilocyte status. Koilocyte-positive cases showed a significantly longer time to regression compared with koilocyte-negative cases (log-rank test, *p* < 0.01). The median time to regression was 38 months in koilocyte-positive cases and 12 months in koilocyte-negative cases. LSIL, low-grade squamous intraepithelial lesion.

The times to regression from LSIL to NILM are illustrated in Figure 3. Kaplan–Meier analysis demonstrated 5-year regression rates of approximately 80% in koilocyte-positive cases and 90% in koilocyte-negative cases. The median (range) time to regression was significantly longer in koilocyte-positive cases (38 [30–45] months) than in koilocyte-negative cases (12 [9–14] months) (log-rank test, *p* < 0.01).

## 4. Discussion

With high-risk HR-HPV testing becoming the primary method of cervical cancer screening, triage strategies are increasingly shifting from cytology-based assessment toward molecular biomarkers. In addition to p16/Ki-67 dual staining [3,4,26], several molecular triage approaches have recently been proposed, including extended HPV genotyping [7], DNA methylation markers [27], and type-specific HPV E6/E7 mRNA testing [28]. These methods may improve risk stratification among HPV-positive women while reducing unnecessary colposcopy referrals. Recent risk-based screening strategies have also incorporated partial HPV genotyping to reduce unnecessary colposcopy referrals among HPV-positive women with low-grade cytological abnormalities [29]. Such approaches further support the need for refined triage strategies beyond morphology alone. This shift reflects the inherent limitations of cytology-based triage in HR-HPV-positive populations. These include its suboptimal performance in identifying CIN2+ lesions and its limited ability to reduce unnecessary colposcopies [3,13,30,31]. CIN2 is a biologically heterogeneous category that includes lesions with a high likelihood of spontaneous regression [32,33]. Consequently, productive HPV-associated lesions and regressive low-grade abnormalities may be included in the target condition, often necessitating the inclusion of ASC-US and LSIL as positive results. This reduces specificity, increases false positives, and ultimately lowers the PPV.

In the present study, most LSIL cases exhibited multiple HPV infections, making it difficult to establish a simple relationship between individual HPV genotypes and koilocytes. Nevertheless, LSIL cases with koilocytes were significantly less likely to be associated with HPV 16/18. Furthermore, consistent with our previous findings [20], we found koilocytosis to represent a genotype-dependent morphological change. Its occurrence was significantly associated with predefined koilocytosis-associated HPV types. However, these associations should be interpreted cautiously and do not imply direct causality. Nevertheless, they suggest that, in populations already confirmed to be HR-HPV-positive, additional cytological evaluation of morphological evidence of HPV infection is of limited clinical value. If koilocytes are primarily associated with HPV types other than HPV 16/18, which are generally considered less likely to persist [15], then koilocytosis has limited utility in defining triage positivity thresholds. It should instead be interpreted as an infection-related finding, regardless of the degree of nuclear atypia. We specifically focused on LSIL rather than ASC-US because LSIL represents a morphologically defined low-grade lesion category in which koilocytosis is more consistently identifiable. In contrast, ASC-US is an equivocal diagnostic category with substantial heterogeneity and limited reproducibility. Restricting the analysis to LSIL allowed for a more focused evaluation of the clinical significance of koilocytosis within HPV-positive low-grade cytological abnormalities.

Koilocytosis has traditionally been regarded as a representative cytological feature of LSIL and used as a basis for its classification [19]. Histological correlation studies have consistently found koilocyte-like changes to be associated with CIN1, supporting the relationship between LSIL and CIN1 [31,34]. However, in the present study, we found that LSIL cases with koilocytes were significantly more likely to correspond to non-CIN lesions. Importantly, this observation was made using cases in which cytology and biopsy were performed concurrently, which minimized any temporal discrepancies. Consistent with our findings, previous studies have reported the presence of koilocytosis in HR-HPV-positive women with low-grade cytology to be a negative predictor of CIN3+. This further supports the limited utility of koilocytosis in identifying clinically significant lesions [35]. Our findings call into question the conventional diagnostic approach, whereby the presence of cells with distinct perinuclear halos leads to LSIL classification based on nuclear atypia. Although the Bethesda system considers HPV-related cellular changes to be features of LSIL [16], the reproducibility and clinical significance of koilocytic atypia interpretations have been questioned [19,36]. Our finding that koilocyte-positive cases are enriched in non-CIN or regressive lesions, whereas koilocyte-negative LSIL cases show a higher prevalence of CIN2+, highlights the heterogeneity of LSIL. It also suggests the need to reconsider the role of koilocytes in risk stratification. These observations do not necessarily challenge the current diagnostic framework, but rather suggest refinement of its interpretation within triage settings. The identified pattern may be explained by the biological heterogeneity of CIN1 and the subjectivity inherent in current CIN1 diagnostic methods. At first glance, associating koilocytosis with low-risk HPV types may seem contradictory. However, because CIN1 encompasses a spectrum of transient HPV-related reactive changes, there is no reason to suppose that koilocytes are indicative of progressive, high-risk HPV-driven lesions. Interobserver variability and differences in diagnostic thresholds further complicate their interpretation. Previous studies have shown that HPV DNA can be detected in lesions that do not meet strict histological criteria, with features such as perinuclear halos and mild nuclear atypia subject to interpretative variability [37,38]. Despite the use of strict criteria, overdiagnosis of koilocytosis and CIN1 has also been reported [38]. Therefore, CIN1 is generally regarded as the histological manifestation of productive HPV infection rather than a true precancerous lesion. The traditional uniform association of koilocytosis with CIN1 may not adequately reflect this heterogeneity.

Another important finding of this study was the incomplete HPV genotype concordance between LBC samples and their corresponding biopsy specimens, regardless of koilocyte status. Specifically, we found cases in which LBC samples were HPV-positive while biopsy specimens were HPV-negative, as well as cases with discordant HPV genotypes. A possible explanation for this is contamination from adjacent anatomical sites, such as the vaginal epithelium, during cervical sampling [19]. This suggests that LBC samples may not exclusively reflect cervical lesions. This may partly explain the higher proportion of non-CIN findings among koilocyte-positive cases in this study. Thus, HPV-infected cells derived from the vaginal epithelium may contribute to LSIL interpretations in cytology without corresponding cervical lesions. Supporting this, single-cell HPV genotyping was concordant with overall LBC samples but showed only moderate (~60%) agreement with the findings from biopsy specimens. Therefore, even when biopsy results are negative, HPV-infected cells from non-cervical sites may be detected in cytology. This underscores the need for cautious interpretation of LSIL findings.

A further finding of this study was that LSIL cases with koilocytes showed significantly longer times to regression to NILM than those without koilocytes. Despite classification based solely on cytoplasmic vacuolization, this difference suggests underlying biological variation between the two groups. We posit that this reflects differences in infection dynamics rather than biological aggressiveness. While this finding may appear to contradict the conclusion that koilocytes have limited utility in triage, koilocytosis is considered a cytological manifestation of productive infection, characterized by active viral replication and particle production [15,39]. This does not necessarily correspond to rapid lesion regression. It may instead reflect a relatively stable phase of persistent infection. Although LSIL generally has a high spontaneous regression rate, the time to regression varies [33,40]. Thus, the delayed regression observed in koilocyte-positive LSIL likely reflects differences in infection dynamics rather than an increased risk of progression. Furthermore, the persistence of koilocytes over a relatively prolonged period increases their likelihood of detection. This may partially explain their high prevalence. Overall, we conclude that koilocytes may indicate infection persistence but have limited utility in predicting CIN2+ or guiding triage decisions. They are more appropriately interpreted as markers of infection.

Strengths of the study include the clinically relevant focus on the role of koilocytosis in HPV-positive women with LSIL cytology, a group in which improved triage may reduce unnecessary colposcopy referrals. The study combines cytology, concurrent histology, HPV genotyping of liquid-based cytology samples and biopsy specimens, microdissection of atypical cells, and clinical follow-up, providing a detailed assessment of the associations between koilocytosis, HPV genotype, histological findings and regression from LSIL to NILM. The use of concurrent biopsy data in most cases strengthens the cytology–histology correlation.

This study had several limitations. First, as a single-center retrospective study, it carries a potential risk of selection bias. In addition, all samples were collected by a single gynecologist at a single institution, which may limit the generalizability of the findings, although this approach reduced variability in sample collection and specimen quality. Second, although HPV genotyping was performed on both cytology and biopsy specimens, the possible contribution of HPV-infected cells from other anatomical sites was not directly assessed, limiting the interpretation of genotype discordance. Third, interobserver variability in the assessment of koilocytosis and LSIL classification may have affected reproducibility. Fourth, the sample size was relatively limited, particularly for subgroup analyses by HPV genotype and histological outcome. Finally, long-term clinical outcomes, including progression to CIN3+, were not fully evaluated. These findings should therefore be interpreted with caution and require validation in larger, multicenter prospective studies.

## 5. Conclusions

Koilocytosis in LSIL appears to reflect productive HPV infection rather than transforming disease and has limited utility for predicting CIN2+ in HPV-based screening triage. Koilocyte-positive lesions are more frequently associated with non-CIN or low-grade HPV-related changes, whereas koilocyte-negative LSIL shows a higher prevalence of CIN2+, highlighting the biological heterogeneity of LSIL.

These findings suggest that excluding infection-related cytological changes, such as koilocytosis, from triage positivity criteria may improve specificity and positive predictive value. However, the potential impact on sensitivity should be carefully considered.

Our results also support the potential value of adopting higher cytology triage thresholds, such as ASC-H or worse, although further prospective validation is required.

The clinically relevant risk of CIN3+ is more likely determined by the underlying HPV genotype and persistence of infection than by koilocytosis or other low-grade morphological abnormalities themselves.

## Figures and Tables

**Table 1:** Cross-tabulation of koilocyte prevalence by human papillomavirus genotype group in patients with low-grade squamous intraepithelial lesions.

		Koilocytes		
		Present	Absent	*p*-Value
		*n* = 93	*n* = 64	
HPV 16/18	Frequencies	12	18	0.017
	Total percentage	40.0%	60.0%	
	Expected frequencies	17.8	12.2	
	Adjusted residual	−2.384	2.384	
Other high-risk types	Frequencies	67	42	0.391
	Total percentage	61.5%	38.5%	
	Expected frequencies	64.6	44.4	
	Adjusted residual	0.858	−0.858	
Non-high-risk HPV types	Frequencies	14	4	0.088
	Total percentage	77.8%	22.2%	
	Expected frequencies	10.7	7.34	
	Adjusted residual	1.701	−1.701	

All *p*-values are two-sided and derived from adjusted standardized residuals. HPV, human papillomavirus.

**Table 2:** Cross-tabulation of koilocyte presence by histological diagnosis in patients with low-grade squamous intraepithelial lesions.

		Koilocytes		
		Present	Absent	*p*-Value
		*n* = 84	*n* = 56	
non-CIN	Frequencies	38	14	0.015
	Total percentage	73.1%	26.9%	
	Expected frequencies	31.2	20.8	
	Adjusted residual	2.428	−2.428	
CIN1	Frequencies	37	26	0.781
	Total percentage	58.7%	41.3%	
	Expected frequencies	37.8	25.2	
	Adjusted residual	−0.277	0.277	
CIN2+	Frequencies	9	16	0.007
	Total percentage	36.0%	64.0%	
	Expected frequencies	15.0	10.0	
	Adjusted residual	−2.703	2.703	

*p*-values are two-sided and are derived from adjusted standardized residuals. CIN, cervical intraepithelial neoplasia.

## Data Availability

The original contributions presented in this study are included in the article/Supplementary Materials. Further inquiries can be directed to the corresponding author.

## Supplementary Materials

The following supporting information can be downloaded at https://www.mdpi.com/article/10.3390/pathogens15050537/s1, Table S1: Clinicopathological and human papillomavirus genotyping data for 157 patients with low-grade squamous intraepithelial lesions.

## Abbreviations

The following abbreviations are used in this manuscript:

ASC-H	Cannot exclude high-grade squamous intraepithelial lesion
ASC-US	Atypical squamous cells of undetermined significance
CI	Confidence intervals
CIN	Cervical intraepithelial neoplasia
DNA	Deoxyribonucleic acid
HSIL	High-grade squamous intraepithelial lesion
LBC	Liquid-based cytology
LSIL	Low-grade squamous intraepithelial lesion
NILM	Negative for intraepithelial lesions or malignancy
PCR	Polymerase chain reactions
PPV	Positive predictive value
