# Supplementary material for: Koilocytosis in LSIL Cytology Has Limited Predictive Value for CIN2+ in HPV-Positive Women: Implications for Risk-Based Cytology Triage
Source: Pathogens. 2026 May 15;15(5):537. doi: 10.3390/pathogens15050537 (PMC13209615; doi:10.3390/pathogens15050537)
Supplement: Supplementary file 1 [file pathogens-15-00537-s001.zip › pathogens-4308697-supplementary.pdf]

Supplementary Table S1. Clinicopathological and human papillomavirus genotyping data for 157 patients with low-grade squamous intraepithelial lesions

Case No.	Koilocyte status	HPV genotypes	HPV genotype groups	Koilocytosis-associated HPV types	HPV genotypes in micro-dissected cells from Pap smear specimens	Simultaneous biopsy	Histological diagnosis	HPV genotypes in micro-dissected biopsy specimens
1	Presence	56	Other high-risk types	Positive	66	Yes	non-CIN	Negative
2	Presence	18, 56, 58, 59, 66, 11, 42, 54, 84	HPV16/18	Positive	56	Yes	CIN1	56, 84
3	Presence	66, 42	Other high-risk types	Positive	66	Yes	CIN1	66
4	Presence	39, 54, 61, 62, 67, 81	Other high-risk types	Positive	not evaluable	Yes	CIN1	67
5	Presence	52	Other high-risk types	Negative	not evaluable	Yes	CIN2	52
6	Presence	53	Non-high-risk HPV types	Positive	53	Yes	CIN1	53
7	Presence	56, 58, 68, 6b, 42	Other high-risk types	Positive	58, 68, 6b	Yes	non-CIN	Negative
8	Presence	56, 58, 6b, 84	Other high-risk types	Positive	not evaluable	Yes	non-CIN	Negative
9	Presence	56, 66	Other high-risk types	Positive	56	Yes	CIN1	56
10	Presence	39, 52, 40, 53, 61	Other high-risk types	Positive	39, 52, 53, 61	Yes	CIN1	52
11	Presence	39, 56, 58	Other high-risk types	Positive	56, 58	Yes	CIN3	58
12	Presence	31, 51, 56, 71, 82	Other high-risk types	Positive	51, 56	Yes	CIN1	56
13	Presence	56, 71, 90	Other high-risk types	Positive	not evaluable	Yes	CIN2	71
14	Presence	39, 56, 59, 62	Other high-risk types	Positive	39	Yes	CIN1	39
15	Presence	16, 51, 56, 58, 68, 62, 82, 90	HPV16/18	Positive	51, 56, 58	No (3 months)	non-CIN	Negative
16	Presence	34, 42, 61, 62, 67, 74	Non-high-risk HPV types	Positive	42, 67	Yes	CIN1	67
17	Presence	52, 56, 58, 6b, 84	Other high-risk types	Positive	6b	Yes	non-CIN	Negative
18	Presence	52, 44, 62, 90	Other high-risk types	Positive	52, 44	Yes	non-CIN	44
19	Presence	39, 58, 55, 74	Other high-risk types	Positive	39	Yes	CIN1	39
20	Presence	31, 56, 81	Other high-risk types	Positive	31	No (15 months)	non-CIN	Negative
21	Presence	53, 54, 55, 74	Non-high-risk HPV types	Positive	53	Yes	non-CIN	Negative
22	Presence	31, 53, 62, 82	Other high-risk types	Positive	53	Yes	CIN2	31, 53, 82
23	Presence	52, 59, 6b, 53	Other high-risk types	Positive	59, 53	Yes	non-CIN	59
24	Presence	56	Other high-risk types	Positive	not evaluable	Yes	non-CIN	90
25	Presence	56, 62	Other high-risk types	Positive	not evaluable	Yes	CIN1	56
26	Presence	56, 71	Other high-risk types	Positive	71	Yes	non-CIN	56
27	Presence	39	Other high-risk types	Positive	39	Yes	CIN1	39
28	Presence	66	Other high-risk types	Positive	66	Yes	non-CIN	66
29	Presence	31, 71, 74	Other high-risk types	Positive	not evaluable	Yes	non-CIN	Negative
30	Presence	30, 40	Non-high-risk HPV types	Positive	not evaluable	Yes	CIN1	40
31	Presence	56, 55, 74	Other high-risk types	Positive	74	Yes	CIN1	56, 74
32	Presence	51, 68, 53, 84, 90	Other high-risk types	Positive	53, 84, 90	Yes	non-CIN	58
33	Presence	51, 56, 6b, 53, 90	Other high-risk types	Positive	not evaluable	Yes	CIN1	53

34	Presence	68, 74	Other high-risk types	Positive	74	Yes	CIN1	68
35	Presence	51, 52, 58, 55, 74	Other high-risk types	Positive	52, 55, 74	Yes	CIN1	52, 74
36	Presence	51, 52, 82	Other high-risk types	Negative	not evaluable	Yes	CIN1	51, 82
37	Presence	39, 56, 73	Other high-risk types	Positive	73	Yes	CIN1	56
38	Presence	39, 53	Other high-risk types	Positive	not evaluable	Yes	non-CIN	Negative
39	Presence	56	Other high-risk types	Positive	not evaluable	No (5 months)	non-CIN	Negative
40	Presence	53	Non-high-risk HPV types	Positive	not evaluable	Yes	CIN1	53
41	Presence	30	Non-high-risk HPV types	Negative	30	Yes	non-CIN	30
42	Presence	33, 39, 56, 59, 68, 40, 42, 73, 81	Other high-risk types	Positive	68	No (31 months)	CIN1	51
43	Presence	56, 62, 74, 81	Other high-risk types	Positive	56	Yes	CIN1	56
44	Presence	51, 82	Other high-risk types	Negative	not evaluable	Yes	CIN1	51
45	Presence	51, 66, 42, 62	Other high-risk types	Positive	not evaluable	Yes	non-CIN	Negative
46	Presence	42, 90	Non-high-risk HPV types	Positive	not evaluable	Yes	non-CIN	42
47	Presence	39, 51, 70, 74, 81	Other high-risk types	Positive	not evaluable	Yes	CIN2	39, 81
48	Presence	52, 59, 42, 90	Other high-risk types	Positive	not evaluable	Yes	non-CIN	90
49	Presence	51, 62, 82, 90	Other high-risk types	Positive	not evaluable	No (6 months)	CIN2	51
50	Presence	33	Other high-risk types	Negative	not evaluable	Yes	CIN1	33
51	Presence	16, 31, 51, 52, 56, 58, 42, 82	HPV16/18	Positive	51	Yes	CIN1	31, 56, 58
52	Presence	56	Other high-risk types	Positive	not evaluable	Yes	non-CIN	Negative
53	Presence	51, 66, 82	Other high-risk types	Positive	not evaluable	Yes	CIN1	51, 66
54	Presence	84, 90	Non-high-risk HPV types	Positive	not evaluable	Yes	non-CIN	84, 90
55	Presence	56, 42, 62	Other high-risk types	Positive	not evaluable	Yes	CIN2	18, 51, 56
56	Presence	56, 58	Other high-risk types	Positive	58	Yes	non-CIN	58
57	Presence	52, 56, 81	Other high-risk types	Positive	not evaluable	Yes	CIN2	45, 52
58	Presence	66	Other high-risk types	Positive	not evaluable	Yes	non-CIN	18, 66
59	Presence	55, 74	Non-high-risk HPV types	Positive	not evaluable	Yes	CIN1	74
60	Presence	16, 39, 56, 59, 66, 74, 90	HPV16/18	Positive	not evaluable	Yes	non-CIN	Negative
61	Presence	16, 51, 52, 59, 74, 82, 90	HPV16/18	Positive	not evaluable	Yes	CIN1	16, 51
62	Presence	53, 74, 90	Non-high-risk HPV types	Positive	not evaluable	Yes	non-CIN	33
63	Presence	52	Other high-risk types	Negative	not evaluable	Yes	CIN1	52
64	Presence	39, 56	Other high-risk types	Positive	not evaluable	No (11 months)	non-CIN	58
65	Presence	16, 56, 59, 66, 74, 90	HPV16/18	Positive	not evaluable	Yes	non-CIN	59
66	Presence	66, 74	Other high-risk types	Positive	not evaluable	Yes	CIN1	52, 66
67	Presence	51, 58, 82	Other high-risk types	Negative	82	Yes	CIN1	18, 51, 58, 82
68	Presence	56, 6b, 74	Other high-risk types	Positive	not evaluable	Yes	non-CIN	6b, 74

69	Presence	16, 52, 56, 55, 74, 81	HPV16/18	Positive	not evaluable	Yes	non-CIN	52
70	Presence	52, 81	Other high-risk types	Negative	not evaluable	No (18 months)	non-CIN	Negative
71	Presence	52, 58	Other high-risk types	Negative	not evaluable	Yes	non-CIN	33, 51
72	Presence	90	Non-high-risk HPV types	Positive	90	Yes	non-CIN	90
73	Presence	45, 59, 66, 53, 62, 67, 81	Other high-risk types	Positive	not evaluable	Yes	non-CIN	45
74	Presence	66	Other high-risk types	Positive	not evaluable	Yes	non-CIN	Negative
75	Presence	42	Non-high-risk HPV types	Positive	not evaluable	Yes	CIN1	42
76	Presence	16, 51, 42, 53, 82	HPV16/18	Positive	not evaluable	Yes	non-CIN	16, 74
77	Presence	16, 56, 74	HPV16/18	Positive	74	No (12 months)	non-CIN	81
78	Presence	16, 66	HPV16/18	Positive	not evaluable	Yes	non-CIN	Negative
79	Presence	66	Other high-risk types	Positive	66	Yes	CIN1	66
80	Presence	51, 58	Other high-risk types	Negative	58	Yes	CIN1	58
81	Presence	39, 51, 56, 58, 42, 53, 82	Other high-risk types	Positive	56	Yes	CIN1	39
82	Presence	51, 70, 82	Other high-risk types	Negative	70	Yes	non-CIN	Negative
83	Presence	42, 61, 62, 71	Non-high-risk HPV types	Positive	not evaluable	Yes	non-CIN	58, 66, 62, 90
84	Presence	53	Non-high-risk HPV types	Positive	not evaluable	Yes	non-CIN	Negative
85	Presence	58	Other high-risk types	Negative	not evaluable	Yes	CIN1	58
86	Presence	16, 56	HPV16/18	Positive	not evaluable	Yes	non-CIN	18
87	Presence	16, 66, 61, 62, 67, 81, 82	HPV16/18	Positive	81	Yes	CIN1	16, 67, 82
88	Presence	56, 68, 34	Other high-risk types	Positive	not evaluable	Yes	non-CIN	Negative
89	Presence	66	Other high-risk types	Positive	not evaluable	Yes	non-CIN	66
90	Presence	66	Other high-risk types	Positive	not evaluable	Yes	CIN2	33, 66
91	Presence	52, 58	Other high-risk types	Negative	not evaluable	No (3 months)	non-CIN	16
92	Presence	56	Other high-risk types	Positive	not evaluable	Yes	CIN2	56
93	Presence	59	Other high-risk types	Negative	not evaluable	Yes	CIN1	59
94	Absence	56, 58	Other high-risk types	Positive	56	Yes	non-CIN	51, 58
95	Absence	58, 66, 53, 82	Other high-risk types	Positive	not evaluable	Yes	CIN2	66
96	Absence	58, 82, 90	Other high-risk types	Positive	not evaluable	Yes	non-CIN	Negative
97	Absence	51, 56, 58, 82, 90	Other high-risk types	Positive	82	Yes	CIN1	Negative
98	Absence	16, 71	HPV16/18	Negative	not evaluable	Yes	CIN1	16
99	Absence	39, 56	Other high-risk types	Positive	39	Yes	CIN1	39, 51
100	Absence	56, 58, 66, 81	Other high-risk types	Positive	66	Yes	CIN1	66
101	Absence	51, 82	Other high-risk types	Negative	not evaluable	Yes	CIN2	82
102	Absence	33, 52, 67	Other high-risk types	Negative	not evaluable	Yes	CIN3	33
103	Absence	56, 58, 42	Other high-risk types	Positive	58, 42	No (7 months)	non-CIN	58
104	Absence	33, 51, 52, 82	Other high-risk types	Negative	51	Yes	CIN1	52

105	Absence	52, 56, 42, 53, 74	Other high-risk types	Positive	52, 42, 53	Yes	CIN1	Negative
106	Absence	52	Other high-risk types	Negative	not evaluable	No (11 months)	non-CIN	Negative
107	Absence	56, 58	Other high-risk types	Positive	not evaluable	Yes	CIN1	58
108	Absence	51, 52, 56, 40, 82	Other high-risk types	Positive	82	Yes	CIN1	Negative
109	Absence	51, 82, 90	Other high-risk types	Positive	not evaluable	Yes	non-CIN	90
110	Absence	18, 39, 56, 62	HPV16/18	Positive	18, 39, 56	Yes	CIN1	18
111	Absence	16, 39	HPV16/18	Positive	39	Yes	CIN3	16
112	Absence	34	Non-high-risk HPV types	Negative	not evaluable	No (5 months)	non-CIN	Negative
113	Absence	66, 53	Other high-risk types	Positive	66	Yes	non-CIN	52, 66
114	Absence	16, 82	HPV16/18	Negative	not evaluable	No (4 months)	non-CIN	Negative
115	Absence	56, 58, 90	Other high-risk types	Positive	not evaluable	Yes	CIN1	58, 90
116	Absence	31, 56, 66, 62	Other high-risk types	Positive	31	Yes	CIN1	31
117	Absence	39, 68	Other high-risk types	Positive	not evaluable	Yes	CIN2	68
118	Absence	51, 56, 61, 82	Other high-risk types	Positive	51, 82	Yes	non-CIN	Negative
119	Absence	39, 56	Other high-risk types	Positive	not evaluable	Yes	CIN1	39
120	Absence	42, 74, 81	Non-high-risk HPV types	Positive	42	No (37 months)	non-CIN	Negative
121	Absence	16, 42	HPV16/18	Positive	not evaluable	Yes	non-CIN	33
122	Absence	56	Other high-risk types	Positive	56	Yes	CIN1	56
123	Absence	53	Non-high-risk HPV types	Positive	not evaluable	Yes	CIN1	53
124	Absence	35, 55, 74	Other high-risk types	Positive	55	Yes	CIN2	35, 55, 74
125	Absence	70	Non-high-risk HPV types	Negative	not evaluable	Yes	CIN1	70
126	Absence	51, 82	Other high-risk types	Negative	not evaluable	Yes	CIN2	51, 82
127	Absence	51, 82	Other high-risk types	Negative	82	Yes	CIN1	51, 82
128	Absence	16, 34	HPV16/18	Negative	not evaluable	Yes	CIN1	16
129	Absence	16, 52	HPV16/18	Negative	not evaluable	Yes	CIN2	52
130	Absence	16, 31, 52, 56, 59, 42, 53, 62, 67, 90	HPV16/18	Positive	not evaluable	Yes	non-CIN	16, 52, 42, 67
131	Absence	52, 42, 67	Other high-risk types	Positive	not evaluable	Yes	CIN2	18, 52.67
132	Absence	16, 56, 74	HPV16/18	Positive	not evaluable	Yes	CIN2	16
133	Absence	16, 39, 56, 58, 53, 74	HPV16/18	Positive	not evaluable	Yes	CIN2	58
134	Absence	52, 34	Other high-risk types	Negative	not evaluable	Yes	non-CIN	59
135	Absence	51, 52, 82	Other high-risk types	Negative	52	Yes	non-CIN	Negative
136	Absence	16, 31, 56	HPV16/18	Positive	not evaluable	Yes	non-CIN	Negative
137	Absence	52, 6b	Other high-risk types	Positive	not evaluable	Yes	CIN1	52
138	Absence	52, 56	Other high-risk types	Positive	not evaluable	Yes	CIN2	52
139	Absence	51, 71, 82	Other high-risk types	Negative	not evaluable	Yes	CIN2	51, 82
140	Absence	16, 66	HPV16/18	Positive	not evaluable	Yes	CIN2	16, 66
141	Absence	51, 82	Other high-risk types	Negative	not evaluable	Yes	CIN1	51, 82
142	Absence	16	HPV16/18	Negative	not evaluable	No (5 months)	CIN2	56

143	Absence	52, 56, 66, 67, 74	Other high-risk types	Positive	not evaluable	Yes	CIN1	45, 52, 67
144	Absence	58, 66, 74	Other high-risk types	Positive	not evaluable	Yes	CIN1	58
145	Absence	52, 82	Other high-risk types	Negative	not evaluable	Yes	CIN2	18
146	Absence	16, 58	HPV16/18	Negative	58	Yes	non-CIN	58
147	Absence	16, 39	HPV16/18	Positive	not evaluable	Yes	CIN1	16, 66
148	Absence	66	Other high-risk types	Positive	not evaluable	Yes	CIN1	66
149	Absence	66	Other high-risk types	Positive	not evaluable	Yes	non-CIN	18
150	Absence	45, 51, 61, 62, 71, 82	Other high-risk types	Positive	82	No (38 months)	non-CIN	16
151	Absence	16	HPV16/18	Negative	not evaluable	Yes	CIN2	16
152	Absence	33, 81	Other high-risk types	Negative	not evaluable	Yes	CIN1	33, 51
153	Absence	52, 53	Other high-risk types	Positive	not evaluable	Yes	non-CIN	53
154	Absence	52, 73	Other high-risk types	Negative	not evaluable	No (13 months)	non-CIN	Negative
155	Absence	16, 39, 45, 52, 58, 71	HPV16/18	Positive	58	Yes	CIN1	16, 39, 45
156	Absence	31, 58	Other high-risk types	Negative	not evaluable	Yes	CIN1	58
157	Absence	18	HPV16/18	Negative	18	Yes	non-CIN	18

HPV, human papillomavirus; CIN, cervical intraepithelial neoplasia
